# Grundlagen für die wissenschaftliche Nutzung umfangreicher Versorgungsdaten in Deutschland – Ergebnisse der AG Data Sharing der Medizininformatik-Initiative

**DOI:** 10.1007/s00103-024-03880-y

**Published:** 2024-04-29

**Authors:** Toralf Kirsten, Philip Kleinert, Marie Gebhardt, Johannes Drepper, Anne-Katrin Andreeff, Fabian Prasser, Oliver Kohlbacher

**Affiliations:** 1 Institut für Medizinische Informatik, Statistik und Epidemiologie, Universität Leipzig, Leipzig, Deutschland; 2 Medizininformatikzentrum, Dept. Medical Data Science, Universitätsklinikum Leipzig, Leipzig, Deutschland; 3 TMF – Technologie- und Methodenplattform für die vernetzte medizinische Forschung e. V., Berlin, Berlin, Deutschland; 4 Institut für Medizinische Informatik und Biometrie, Medizinische Fakultät Carl Gustav Carus der Technischen Universität Dresden, Dresden, Deutschland; 5 Center of Health Data Science, Medizininformatik, Berliner Institut für Gesundheitsforschung in der Charité – Universitätsmedizin Berlin, Berlin, Deutschland; 6 Institut für Biomedizinische Informatik, Universität Tübingen, Sand 14, 72074 Tübingen, Deutschland; 7 Fachbereich Informatik, Universität Tübingen, Tübingen, Deutschland; 8 Institut für Translationale Bioinformatik, Universitätsklinikum Tübingen, Tübingen, Deutschland

**Keywords:** Medizininformatik, Datenaustausch, Real-World-Daten, Universitätsmedizin, Multizentrische klinische Forschung, Medical informatics, Data sharing, Real-world data, University medicine, Multicentric clinical research

## Abstract

Versorgungsdaten stellen eine wichtige Ressource in der angewandten medizinischen Forschung dar. Sie liegen multizentrisch vor. Es bleibt jedoch eine Herausforderung, standardisierte Datenaustauschprozesse zwischen Bundesländern und ihren individuellen Gesetzen und Vorschriften zu ermöglichen. Die Medizininformatik-Initiative (MII) wurde im Jahr 2016 gegründet, um Prozesse zu implementieren, die einen klinikübergreifenden Zugriff auf Versorgungsdaten in Deutschland ermöglichen. Mehrere eingerichtete Arbeitsgruppen konzipieren standardisierte Datenstrukturen (AG Interoperabilität), Patienteninformationen und Einwilligungserklärungen (AG Consent) sowie Regelungen zum Datenaustausch (AG Data Sharing). Hier stellen wir die wichtigsten Ergebnisse der Arbeitsgruppe Data Sharing vor, die unter anderem abgestimmte Nutzungsbedingungen, rechtliche Regelung und Datenzugriffsprozesse enthalten. Diese werden bereits von den etablierten „Datenintegrationszentren“ (DIZ) und „Use and Access Committees“ (UACs) umgesetzt. Wir beschreiben die Dienste, die notwendig sind, um Forschenden einen standardisierten Datenzugang zu ermöglichen. Sie werden u. a. mit dem Forschungsdatenportal für Gesundheit (FDPG) umgesetzt. Auf dieser Basis wurden seit der Pilotphase die Prozesse von 406 aktiven Forschenden verwendet, was zum Stand April 2024 zu 19 registrierten Projekten und 31 eingereichten Forschungsanträgen geführt hat.

## Abstract (en)

Healthcare data are an important resource in applied medical research. They are available multicentrically. However, it remains a challenge to enable standardized data exchange processes between federal states and their individual laws and regulations. The Medical Informatics Initiative (MII) was founded in 2016 to implement processes that enable cross-clinic access to healthcare data in Germany. Several working groups (WGs) have been set up to coordinate standardized data structures (WG Interoperability), patient information and declarations of consent (WG Consent), and regulations on data exchange (WG Data Sharing). Here we present the most important results of the Data Sharing working group, which include agreed terms of use, legal regulations, and data access processes. They are already being implemented by the established Data Integration Centers (DIZ) and Use and Access Committees (UACs). We describe the services that are necessary to provide researchers with standardized data access. They are implemented with the Research Data Portal for Health, among others. Since the pilot phase, the processes of 385 active researchers have been used on this basis, which, as of April 2024, has resulted in 19 registered projects and 31 submitted research applications.

## Einleitung

Klinische Daten zu gesundheitlichen Aspekten bilden die Grundlage für die angewandte medizinische Forschung. Sie werden in all ihrer Vielfalt in verschiedenen Institutionen und für verschiedene Zwecke aufgenommen und eingesetzt.

Klinische Studien fokussieren hauptsächlich auf klinische Fragestellungen, z. B. die Wirksamkeit eines neuen Medikaments, einer neuen Therapie oder eines angepassten Therapie-Settings. Dagegen zielen epidemiologische Studien oftmals auf die Erhebung der Prävalenzen von ausgewählten Erkrankungen in der Bevölkerung ab. Gezielte medizinische Studien haben oftmals sehr eingeschränkte Zielstellungen, sodass die dafür erhobenen Daten in ihrer Aussagekraft limitiert sind. Die Routinedaten, die während der Aufenthalte von Patientinnen und Patienten in Kliniken erhoben werden, dienen primär der Dokumentation des Arzt-Patienten-Kontakts in Hinsicht auf die ausgeführte Diagnostik und die durchgeführten Behandlungen (Therapien). Gleichwohl stellen sie eine fundierte Datenquelle für die medizinische Forschung dar, auch wenn ihr primärer Zweck sich von dem der o. g. Studien unterscheidet. Routinedaten aus der gelebten klinischen Praxis sind umfangreicher, heterogener und deutlich komplexer als Studiendaten.

Routinedaten aus den Dokumentationssystemen der Krankenhäuser sind meist umfangreicher und aussagekräftiger als Krankenkassendaten, die zudem aufgrund ihres Zwecks der Abrechnung erbrachter Leistungen nicht repräsentativ sind. Letztlich erfordert die medizinische Forschung den Vergleich und die Zusammenschau unterschiedlicher Datenquellen.

Die Forschung mit klinischen Routinedaten kann der beständigen Verbesserung der Patientensicherheit, der schnelleren Diagnostik und Behandlung und damit einem erstrebenswerten Gemeinwohl dienen. In vielen Fällen sind multizentrische Auswertungen in der Lage, die nötigen Fallzahlen für eine statistisch robuste Evidenzgenerierung zu ermöglichen. Die Verwendung klinischer Routinedaten für medizinische Forschungszwecke über Klinikbereiche, Standorte und Bundesländergrenzen hinweg bedarf jedoch einer Harmonisierung. Da es sich um personenbezogene Gesundheitsdaten handelt, die besonders schützenswert sind, gelten besondere Bedingungen für die Nutzung, so gibt es verschiedene europäische, landes- und bundeslandspezifische Regelungen zum Datenschutz, was Auswirkungen auf die Nutzung der Daten hat. Erklärtes Ziel der im Jahr 2016 gegründeten Medizininformatik-Initiative (MII) ist es, den Konflikt zwischen vollständigem Datenschutz und bestmöglicher Nutzung von Daten zur medizinischen Forschung zu lösen und bundeslandübergreifend in den deutschen Universitätskliniken umzusetzen. Datenintegrationszentren (DIZ) werden aufgebaut, um Forschenden den Zugriff auf die klinischen Routinedaten zu ermöglichen. Die Arbeitsgruppe *Data Sharing*, als Teil einer übergreifenden Koordinierungsstruktur innerhalb der MII, hat zum Ziel, organisatorische Grundlagen für das deutschlandweite Teilen und Nutzen der Daten zu schaffen.

Das Teilen von Daten ist oft mit hohen technischen, rechtlichen, aber auch kulturellen Hürden verbunden. Patientinnen und Patienten unterstützen die Nutzung ihrer Daten für die Forschung überwiegend, sofern entsprechende Maßnahmen zum Schutz ihrer Daten gegeben sind [2]. Die AG Data Sharing hat Prozesse und Methoden mitgestaltet und etabliert, die multizentrisches Datenteilen sicher und effizient ermöglichen und gleichzeitig die Bedenken gegen die Datennutzung berücksichtigen.

Die FAIR-Prinzipien [1] bilden die Grundlage für das heutige Data Sharing. Die 4 Buchstaben von FAIR stehen für die Anforderungen Auffindbarkeit („findable“), Zugriff („accessible“), Interoperabilität („interoperable“) und Wiederverwendbarkeit („reusable“). Während die Interoperabilität ein Grundanliegen der Arbeitsgruppe (AG) Interoperabilität in der MII-Koordinierungsstruktur ist, obliegen die Auffindbarkeit, der Zugriff und die Regelungen zur Wiederverwendung der AG Data Sharing, wobei die Wiederverwendung zum Teil auch von der AG Consent bearbeitet wird, die sich der informierten, breiten Einwilligung der Patientinnen und Patienten widmet.

Mit diesem Artikel geben wir einen Überblick über die Struktur und Arbeitsweise der AG Data Sharing und fassen die wichtigsten Ergebnisse der AG im Rahmen der MII zusammen. Insbesondere steht die klinikübergreifende Datennutzung im Fokus, also die integrierte Nutzung von Daten mehrerer Kliniken.

## Struktur und Arbeitsweise der AG Data Sharing

Die AG Data Sharing wurde mit Beginn der Vorbereitungs- und Konzeptionsphase der MII im Jahr 2017 vom Nationalen Steuerungsgremium (NSG) einberufen. Sie hat die Aufgabe, übergreifende Aspekte des Teilens medizinischer Daten aus Universitätskliniken zu diskutieren und abzustimmen. Die AG ist in den Verbund verschiedener AGs eingebettet, zu denen die AG Consent, die AG Interoperabilität, die AG Kommunikation und die AG externe Daten gehören. Diese AGs berichten ihre Ergebnisse dem NSG, welches diese beschließen kann und sie damit für die in der MII vertretenen Universitätskliniken verbindlich macht.^1^

Teil der AG Data Sharing sind sogenannte Task-Forces (TFs), die einzelne Themen im Detail diskutieren und bearbeiten. Dies sind derzeit die TFs „Use & Access“, „Widerruf“, „Prozessmodelle“, „Audit“ und „Verteilte Analysen“, die im Folgenden vorgestellt werden.

Die *TF Use & Access* widmet sich der Aufgabe, organisatorische Regelungen für einen standortübergreifenden Datenzugang zu konzipieren und zu etablieren und in einer Nutzungsordnung zu fassen. Dazu trägt langjährige Expertise aus Zentren bei, an denen große epidemiologische Studien durchgeführt werden, darunter insbesondere die Universität Greifswald mit den SHIP-Studien [3] sowie die Universität Leipzig mit dem LIFE – Leipziger Forschungszentrum für Zivilisationserkrankungen und den dort durchgeführten Studien (z. B. LIFE-Adult [4]). Als konzeptionelle Grundlage diente das „Open Archive Information System“ (OAIS [5]), das die 3 am Data-Sharing-Prozess beteiligten Strukturen und deren Aufgaben benennt: den Datenproduzenten („data producer“), den Datenkonsumenten („data consumer“) sowie die datenverwaltende Stelle.

Im Fokus der *TF Prozessmodelle* stehen die Prozesse des übergreifenden Data Sharing, die sich von der Vorbereitung und Planung bis hin zum abschließenden Datenaustausch erstrecken. Auf Basis der Prozessmodelle begann die Arbeit an deren erweiterten Versionen, in denen insbesondere Teilprozesse identifiziert und harmonisiert wurden, sodass diese standardisiert ablaufen können. Insbesondere wurden die Prozessmodelle mit der von der TF Use & Access entworfenen Nutzungsordnung beständig abgeglichen. Die Prozessmodelle wurden in der „Business Process Modelling Notation“ (BPMN^2^) in Abstimmung mit lokalen Expertinnen und Experten aufgezeichnet.

Um den Fortgang der Aufbauarbeiten und damit den Zielerreichungsgrad an den Datenintegrationszentren zeitnah und regelmäßig überprüfen zu können, wurden von der *TF Audit* geeignete Qualitätsindikatoren geschaffen. Dazu wurde die Expertise aus dem Netzwerk der Koordinierungszentren für Klinische Studien (KKS) herangezogen. Diese Zentren unterliegen regelmäßigen Audits, d. h. Prüfungen, die von einem bestellten unabhängigen Prüfinstitut durchgeführt werden. Die mit der TF Audit geschaffenen Ergebnisse waren Grundlage für „Friendly Audits“ (Voraudits zur Feststellung der Zertifizierungsfähigkeit) an beinahe allen Standorten sowie für die Vorbereitung des Auditierungsprozesses, der 2021 vor Ende der ersten Förderperiode an den Datenintegrationszentren aller Konsortien von einem extern bestellten Prüfer durchgeführt wurde.

Die *TF Verteilte Analysen* arbeitet an Methoden und Strukturen, die eine standortübergreifende, dezentrale Analyse ermöglichen. Dazu wurden Infrastrukturen wie DataSHIELD [6], das Personal-Health-Train-Konzept [7, 8] und verschiedene Lösungen unter Nutzung von Secure Multi Party Computation (SMPC; [9–11]) untersucht. Eine Datenherausgabe ausschließlich in materieller Form würde für die Forschenden zu kurz greifen. Wissenschaftliches Personal kann bereits im Sinne der Eigenforschung (abhängig von den gesetzlichen Grundlagen je Bundesland) lokal am eigenen Klinikum auf diese Daten (ohne Einwilligung der Patientinnen und Patienten) zugreifen. Eine zentrale Datenverwaltung und -analyse ist jedoch nur mit Daten von Patientinnen und Patienten möglich, die ihre Einwilligung dazu erteilt haben.

Die *TF Widerruf* befasst sich mit dem Verfahren zur Berücksichtigung von Widerrufen der Einwilligung von Patienten oder Patientinnen in die Nutzung ihrer Daten für die Forschung. Der Prozess berücksichtigt die Rechte der Patientinnen und Patienten bei lokalen wie auch übergreifenden Forschungsprojekten.

## Ergebnisse der AG Data Sharing

In der AG Data Sharing und den etablierten Task-Forces wurde eine Vielzahl an Ergebnissen erzielt, von denen an dieser Stelle nur eine Auswahl wiedergegeben und kurz charakterisiert werden kann.

### Prozesse zur Datennutzung

Die medizinischen Behandlungsdaten der Universitätskliniken sind personalisiert. Forschende können deshalb nur unter Wahrung des Datenschutzes, also unter Beachtung bestimmter Restriktionen und Bedingungen, auf die Daten zugreifen. Die im Rahmen der MII geschaffenen Datenintegrationszentren (DIZ) der Standorte bereiten die Daten interoperabel (basierend auf Spezifikationen der AG Interoperabilität) auf und stellen erste Datenschutzmaßnahmen sicher (z. B. eine Pseudonymisierung der Daten). Außerdem verwalten sie den Zugriff auf die Daten gemäß dem OAIS-Konzept als Transferstelle. Zur Verpflichtung auf die von der AG Data Sharing entworfene *Nutzungsordnung*^3^ und zur Prüfung der Einhaltung der Restriktionen sind alle Daten vor der Datennutzung zu beantragen. Der *Datennutzungsantrag*^4^ wurde von der AG spezifiziert und abgestimmt.

**Abb. 1:**
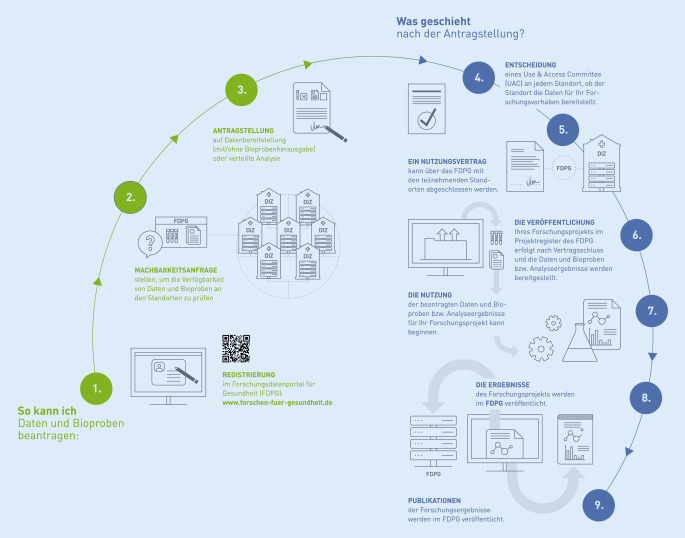
Prozess der Datennutzung von der Registrierung und Beantragung bis zur Publikation. (Quelle: TMF e. V.)

Der *Datennutzungsvertrag*^5^ wurde von der AG zur schnellen Bearbeitung standardisiert und abgestimmt. Abb. 1 gibt eine Übersicht über den vereinheitlichten Antragsprozess, der für die Forschenden grafisch aufbereitet wurde.

### Informationsaufbereitung und Darstellung der verfügbaren Daten für Forschende

Die Forschenden sollten vor der Datenbeantragung genau recherchieren, welche Daten verfügbar sind. Dazu wurde von der AG Data Sharing spezifiziert, welche Einsichtnahme in verfügbare Daten hierfür benötigt wird. Die AG unterscheidet 4 Stufen des Data Sharing, von denen sich die ersten beiden auf Informationen zum Datenmodell und zu den verfügbaren Daten beziehen.

Die erste Stufe beinhaltet ausschließlich Informationen zum Datenmodell. Innerhalb der MII hat man sich darauf geeinigt, alle strukturierten medizinischen Daten nach dem FHIR-Standard (Fast Healthcare Interoperable Resources)^6^ aufzubereiten. FHIR und seine Adaption für die Datenintegrationszentren sind frei zugänglich.^7^

**Abb. 2:**
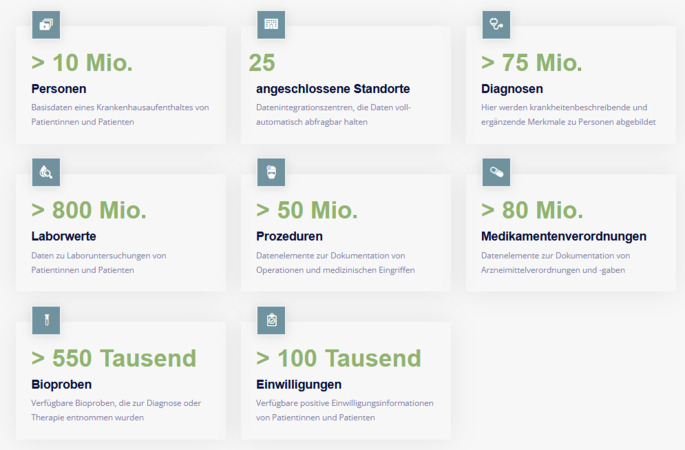
Das „Schaufenster“ des Forschungsdatenportals für Gesundheit (FDPG) zeigt übersichtlich an, welche Kategorien an Daten in welcher Gesamthäufigkeit an den teilnehmenden Universitätskliniken verfügbar sind. (Quelle: forschen-fuer-gesundheit.de; zugegriffen am 10.01.2024)

Die zweite Stufe zielt auf die aggregierte Deskription von Datenbeständen. Darunter werden in erster Linie die Häufigkeiten ausgewählter Datenitems verstanden, z. B. die Anzahl von Patientinnen und Patienten oder die Anzahl von Diagnosen, Prozeduren und Laborwerten. Diese deskriptiven Daten werden in einem „Schaufenster“ öffentlich ohne Einschränkung des Zugriffs zur Verfügung gestellt und regelmäßig aktualisiert (Abb. 2).

### Machbarkeitsanfragen und Datenbeantragung mit dem Forschungsdatenportal für Gesundheit

Mit der dritten Stufe des Data Sharing sollen Forschende die Möglichkeit haben, konkrete Anfragen zum Datenbestand^8^ zu formulieren. Dahinter steht die Frage, ob der verfügbare Datenbestand genügend Patientinnen und Patienten umfasst, um die konkrete Forschungsfragestellung adressieren zu können. Dazu wurde das Forschungsdatenportal für Gesundheit (FDPG) etabliert, in dem Forschende Ein- und Ausschlusskriterien spezifizieren und vollautomatisch Abfragen an angeschlossene Standorte schicken können, um die Machbarkeit der Studie vor Beantragung zu überprüfen. Die Kriterien (z. B. Geschlecht = „weiblich“) können mit logischen Operatoren AND und OR verbunden werden [12].

Für derart ausgeführte Machbarkeitsabfragen wurden restriktive Datenschutzmaßnahmen umgesetzt. Dazu gehören die Limitierung der Anzahl an Abfragen sowie das Verrauschen der ermittelten Häufigkeit von Patientinnen und Patienten am Klinikum. Geringe Häufigkeiten sowohl von Patientinnen und Patienten als auch von Kliniken werden nicht angezeigt, um das Risiko einer Reidentifizierung weiter zu verringern. Die Kliniken werden dabei verschleiert (nur als „Klinikum 1“ usw. gekennzeichnet), sodass kein Rückschluss auf einzelne Universitätskliniken genommen werden kann. Der Zugang zu den Machbarkeitsabfragen ist nicht öffentlich; er setzt eine Forschungstätigkeit sowie Registrierung voraus.

Im Gegensatz zu den 3 vorgenannten Stufen des Data Sharing, in denen Metadaten ausgetauscht wurden, bezieht sich die vierte Stufe auf das Teilen der medizinischen Daten selbst. Dazu müssen Forschende den Antragsprozess vollständig durchlaufen. Als technische und organisatorische Basis dient das FDPG, in dem Informationen und Prozesse zur Datennutzung gebündelt werden.

Im Antrag enthalten sind die wissenschaftliche Zielstellung, ein Analyseplan, die Machbarkeitsanfrage sowie die zum Erreichen der Ziele notwendigen Variablen. Darüber hinaus erfordert jeder Datennutzungsantrag ein Ethikvotum, das zusammen mit dem eingereichten Datennutzungsantrag an alle angebundenen Datenintegrationszentren übermittelt wird. An jedem DIZ werden die Datennutzungsanträge von einem Use & Access Committee (UAC) geprüft. Im Ergebnis melden die DIZ die Zustimmung oder die begründete Ablehnung über das FDPG zurück. Rückfragen werden im Portal zentral koordiniert.

Im Anschluss an die Beantragung und Begutachtung wird ein Vertrag zwischen den datengebenden Standorten und der Institution des Antragsstellenden geschlossen. Zeitgleich wird das Projekt im Projektregister^9^ des FDPG veröffentlicht. Das Projektregister fungiert als öffentlich zugängliches Transparenzportal; damit können sowohl Patientinnen und Patienten, Forschende als auch die interessierte Öffentlichkeit einen Überblick bekommen, welche Projekte durchgeführt werden oder wurden. Die datengebenden Einrichtungen und die beantragende Person werden aufgeführt. Letztere ist die Ansprechperson für weitere Nachfragen, z. B. in Hinblick auf gemeinsame Forschungsprojekte.

### Vorlagen für die Beantragung von Daten und Bioproben und die Vertragsgestaltung

Die Beantragung von Daten und Bioproben aus verschiedenen Universitätskliniken wird umso einfacher, je mehr die Prozesse und die benötigten Daten harmonisiert und standardisiert sind. Dies schließt ebenso die Bereitstellung der Vorlage eines Datennutzungsvertrags mit allen notwendigen Anlagen ein, die mit allen Justiziariaten der an der MII teilnehmenden Universitätskliniken diskutiert und abgestimmt wurde; ein konkreter Datennutzungsvertrag bedarf nur noch der Aufnahme aller Partner und eventuell erteilter Auflagen sowie als Anlage des Datennutzungsantrags. Damit vereinfacht und verkürzt sich der Antragsprozess bis zum Vertragsschluss, insbesondere wenn viele Partner inkludiert sind. Breit geprüfte Vorlagen für den Nutzungsvertrag sind auf den Webseiten der MII verfügbar.^10^

### Übergreifende Nutzungsordnung als Vorlage für lokale Nutzungs- und Geschäftsordnungen

Grundlage des Handelns der Datenintegrationszentren bezogen auf die lokalen und übergreifenden Datennutzungsprojekte sind vorab verbindlich getroffene Regelungen und Verfahrensweisen. Damit alle Datenintegrationszentren nach denselben Grundsätzen handeln, ist eine Harmonisierung der Regelungen in diesen Geschäftsordnungen notwendig. Dazu wurde eine übergreifende Nutzungsordnung^11^ konzipiert und abgestimmt. Diese definiert den grundsätzlichen Antragsprozess (s. oben) und alle Folgeprozesse und benennt die regulatorischen Grundlagen sowie die Rechte und Pflichten der am Prozess Beteiligten. Darüber hinaus gibt sie Empfehlungen zum Umgang mit Abweichungen (z. B. Ergänzungen in Studienprotokollen). Lokale Nutzungs- oder Geschäftsordnungen können darauf Bezug nehmen und Erweiterungen mit lokalspezifischen Regelungen vornehmen.

Alle Vereinbarungen, die für die Durchführung von Datennutzungsprojekten zwischen den Standorten der MII erforderlich sind, werden im Teilnahmerahmenvertrag der MII getroffen. Alle vorgenannten Bausteine (Datennutzungsantrag, Nutzungsordnung, Standardnutzungsvertrag und Teilnahmerahmenvertrag) ergeben zusammen das MII-Vertragswerk, das gemeinsam mit allen organisatorischen und technischen Komponenten der MII stetig weiterentwickelt und optimiert wird.

### Strukturen zur Durchführung von verteilten Analysen

Aufgrund der föderalen Datenhaltung und Datenschutzbestimmungen wurden innerhalb der MII frühzeitig verschiedene Ansätze und Strukturen zur verteilten Analyse bearbeitet. Im Gegensatz zur zentralen Datenzusammenführung („Bringe die Daten zur Analyse“) verlassen bei der verteilten Analyse die Daten den Standort nicht („Bringe die Analyse zu den Daten“). Damit wird die Souveränität des Datengebenden in Bezug auf seine Daten gewahrt. Es muss gewährleistet werden, dass die Daten an den datengebenden Einrichtungen verbleiben und dort verarbeitet (analysiert) werden.

Neben dem Ausführen von geprüften, öffentlich einsehbaren Skripten zur Analyse der Projektdaten am Standort pilotiert die MII Prozesse und Rahmenbedingungen für verteilte Analysen mit der Plattform DataSHIELD^12^. Dazu wurde begleitend ein Dokumentationskonzept entworfen, das verschiedene Dokumentationen in sich vereint, die zum Betrieb der Verteilten-Analyse-Infrastruktur notwendig sind. Es ist generisch, sodass die aktuelle Dokumentation auf weitere Infrastrukturen übertragen werden kann.

Aufbauend auf den Erfahrungen, die mit den Infrastrukturen zur verteilten Analyse von medizinischen Daten gemacht wurden, zu denen sowohl DataSHIELD als auch verschiedene Implementierungen des Konzepts „Personal Health Train“ (PHT) zählen, wird mit dem Projekt PrivateAIM^13^ in der aktuellen Förderphase der MII eine Implementierung entlang der Anforderungen der Datenintegrationszentren vorgenommen. Neben dem grundlegenden Ziel, so datenschutzkonformes maschinelles Lernen und Datenanalytik innerhalb der Plattform der MII zu ermöglichen, möchte das Projekt auch maßgeschneiderte KI-Methoden, Föderationsmechanismen und „Privacy-Enhancing-Technologies“ für die medizinische Datenanalytik der Zukunft entwickeln.

### Qualitätsindikatoren und -prozesse für den Aufbau von Datenintegrationszentren

Letztlich bedarf es einer beständigen Kontrolle und Steuerung, inwieweit die im Aufbau befindlichen Strukturen und Prozesse zu einer Unterstützung des Data Sharing beitragen. Dazu wurden von der TF Audit Indikatoren festgelegt, die sich grob in die Kategorien Struktur‑, Prozess- und Ergebnisqualität einteilen lassen. Zu vielen dieser Prozesse wurden Standard Operating Procedures (Standardverfahrensanweisungen, SOP) an den Datenintegrationszentren etabliert, zu denen die Mitarbeitenden geschult und beständig trainiert werden. Indikatoren der Ergebnisqualität richten sich auf die erzielten Ergebnisse eines Datenintegrationszentrums, z. B. die Möglichkeiten des DIZ, die unterschiedlichen Formen des Datenzugriffs (Datenherausgabe vs. verteilte Analyse) zu bedienen. Entlang dieser Indikatoren wurden schon während der ersten Förderperiode Begutachtungen durchgeführt. Eine externe Begutachtung fand zum Ende der ersten Förderperiode durch eine unabhängige Institution statt. Im Frühjahr 2021 wurden als Ergebnis des komplexen Auditierungsverfahrens 90 % der Standorte als Datenintegrationszentren „im fortgeschrittenen Aufbaustadium“ bewertet.

### Durchgeführte Datennutzungsprojekte

In der Pilotphase wurde eine Reihe von Datennutzungsprojekten in Form von „Projectathons“ initiiert und durchgeführt [13]. Im Bereich der Kardiologie wurden Analysen zu NT-proBNP^14^-Werten bei Vorhofflimmern umgesetzt und die unterschiedlichen technischen Aspekte der MII-Prozesse evaluiert: (1) zentrale Datenzusammenführung, (2) verteilte Analyse mit Skripten und (3) verteilte Analyse mittels DataSHIELD. Die Ergebnisse wurden aufgearbeitet und verbessern die etablierten Prozesse kontinuierlich [13]. Das neurologische Projekt WE-STORM zielt auf die Entwicklung und Validierung statistischer und maschineller Lernmodelle zur Vorhersage von Schlaganfallhäufigkeiten unter Einbeziehung meteorologischer und klinischer Risikofaktoren ab. Im Rahmen des endokrinologischen und diabetologischen Projekts DiaClusT werden Subphänotypen von Typ-2-Diabetes untersucht, um personalisierte Behandlungsansätze zu entwickeln. POLAR-DABI fokussiert auf nephrologische und pharmakologische Fragestellungen. Untersucht wird die Verordnungspraxis des Antikoagulans Dabigatran und seine Auswirkung auf die Nierenfunktion. Unter dem Dach von CORD-MI werden Studien zu Mukoviszidose, Phenylketonurie, zum Kawasaki-Syndrom und PIMS (Pediatric Inflammatory Multisystem Syndrome) sowie zu weiteren ausgewählten Seltenen Erkrankungen bearbeitet und Datenqualitätsprüfungen durchgeführt. Neben diesen standortübergreifenden Projekten wurden auch lokale Datennutzungsprojekte mittels der MII-Strukturen abgestimmt. Beispiele umfassen ein Register für Patienten mit hepatobiliären Systemtumoren sowie regelmäßige Machbarkeitsanfragen für multizentrische Forschungsprojekte.

Das FDPG steht seit dem 16.05.2023 allen Forschenden zur Verfügung und ist nicht mehr auf MII-interne Projekte eingeschränkt. Stand April 2024 nutzen 405 aktive Forschende das Portal. Mehr als 10.000 Machbarkeitsanfragen wurden durchgeführt und automatisiert von bis zu 25 angeschlossenen Standorten beantwortet. 19 Projekte sind bereits gestartet und wurden im Transparenzregister veröffentlicht. Ein Projekt ist vollständig abgeschlossen. 12 weitere Projekte wurden beantragt und durchlaufen die etablierten Prozesse (vor der Veröffentlichung im Register) aktuell.

## Diskussion und Schlussfolgerungen

Die Medizininformatik-Initiative (MII) ermöglicht Zugriff auf standortübergreifende klinische Routinedaten durch Standardisierung der Datenhaltung und Datenbeantragung. Die AG Data Sharing hat hier entscheidend zu Entwicklung und Aufbau effizienter Prozesse beigetragen, die heute multizentrische Datennutzung erlauben und weiter ausbauen.

Mit dem Aufbau von Datenintegrationszentren an deutschen Universitätskliniken ist ein Meilenstein zur Unterstützung der medizinischen Forschung gelungen. Die Datenintegrationszentren stellen den einheitlichen Zugang zu den Daten in den Universitätskliniken dar. Sie werden die Digitalisierung und den Auf- und Ausbau von interoperablen Informationssystemen in den Universitätskliniken in den nächsten Jahren vorantreiben. Dabei werden sie stets die mit steigender Nutzungszahl immer häufigeren Anfragen auf noch unerschlossene oder nicht digital vorliegende Daten berücksichtigen. Der Fokus der MII auf die Universitätskliniken war für den ersten Schritt von Vorteil; sie sind ausnahmslos Maximalversorger mit einem großen Patientenaufkommen und damit einem hohen verfügbaren Anteil an Gesundheitsdaten. In nächster Zeit sollen auch kleinere Kliniken und andere Gesundheitsdienstleister sukzessive in die aufgebauten übergreifenden Infrastrukturen zum Data Sharing inkludiert werden.

Die Qualität von Routinedaten aus der medizinischen Versorgung kann mäßig sein, da diese Daten nicht primär zum Zwecke der Forschung erhoben werden. Die standardisierten Prozesse der Datenhaltung (AG Interoperabilität), aber insbesondere auch die der AG Data Sharing tragen dazu bei, die Datenerfassung zu verbessern und damit auch die Qualität der Daten für die Versorgung und Forschung zu erhöhen. Dies ist notwendig, um aussagekräftige evidenzbasierte Forschung (unter anderem auch mit maschinellem Lernen) an den immens umfangreichen Datensätzen der Routineversorgung durchführen zu können.

Im Fokus der standardisierten Prozesse befindet sich stets auch die Verbesserung der Versorgung von Patientinnen und Patienten. Diese profitieren von der Forschung mit Routinedaten nicht nur durch verbesserte Therapiemöglichkeiten, sondern auch durch die verbesserten Prozesse in der Versorgung selbst, die durch die Vereinheitlichung und Standardisierung, wie zum Beispiel der Datenerfassung, ermöglicht werden.

Ungeklärt ist, wie diese Gesundheitsdateninfrastruktur dauerhaft finanziert wird; Betrieb und Wartung von IT-Infrastrukturen sowie organisatorische und vertragliche Administration von Projekten kosten Geld und bedürfen daher einer nachhaltigen Ausfinanzierung.

Obwohl die etablierten Data-Sharing-Prozesse bereits produktiv genutzt werden, bedarf es weiterer Optimierung und Verschlankung, um kürzere Beantragungszeiten bis zur Erteilung des Datenzugriffs für Forschende zu erreichen. Dies inkludiert den weiteren Auf- und Ausbau von Infrastrukturen, die im Kleinen (z. B. elektronische Vertragsunterzeichnung) wie im Großen wirken. Für Letzteres werden in der aktuellen Förderperiode verschiedene Projekte, sogenannte Methodenplattformen (z. B. PrivateAIM, GemTex), gefördert. Dadurch wird die Nutzung der aufgebauten Infrastrukturen weiter vorangetrieben, die Forschungsgemeinschaft und somit die medizinische Forschung werden gestärkt.

Letztlich bleibt festzuhalten, dass auf der Grundlage der aufgebauten Infrastrukturen und Regelungen ein Zugang zu FAIR-Daten in der medizinischen Forschung möglich ist. Sie sind eine ideale Ausgangsbasis für die Einbindung und Verknüpfung von Routinedaten mit weiteren verfügbaren Datenquellen. Studiendaten, Kassendaten und Registerdaten, wie z. B. Krebs-Registerdaten, sind erweiternde Daten, die die medizinische Forschung auf Routinedaten bereichern. Die Zusammenarbeit mit diesen Dateninfrastrukturen muss stetig verbessert werden. Die etablierten Prozesse der AG Data Sharing ermöglichen dies nun, sodass Deutschland eine gute Ausgangsbasis geschaffen hat, um in den europäischen Datenraum einzutreten.

## Footnotes

1 https://www.medizininformatik-initiative.de/de/geschaeftsordnung-des-nationalen-steuerungsgremiums-der-deutschen-medizininformatik-initiative. Zugegriffen am 20.12.2023.

2 https://www.omg.org/spec/BPMN. Zugegriffen am 20.12.2023.

3 https://www.medizininformatik-initiative.de/de/nutzungsordnung. Zugegriffen am 20.12.2023.

4 https://www.medizininformatik-initiative.de/sites/default/files/2020-12/DatNutzAntr_v1.0.pdf. Zugegriffen am 20.12.2023.

5 https://www.medizininformatik-initiative.de/de/nutzungsvertrag. Zugegriffen am 20.12.2023.

6 https://www.hl7.org/fhir/overview.html. Zugegriffen am 20.12.2023.

7 https://www.medizininformatik-initiative.de/de/der-kerndatensatz-der-medizininformatik-initiative. Zugegriffen am 20.12.2023.

8 Der im Folgenden skizzierte Beantragungsprozess für Daten gilt mit kleinen Unterschieden ebenso für Bioproben.

9 https://forschen-fuer-gesundheit.de/menu_register.php. Zugegriffen am 20.12.2023.

10 https://www.medizininformatik-initiative.de/de/nutzungsvertrag. Zugegriffen am 20.12.2023.

11 https://www.medizininformatik-initiative.de/de/nutzungsordnung. Zugegriffen am 20.12.2023.

12 https://www.datashield.org. Zugegriffen am 20.12.2023.

13 https://privateaim.de/. Zugegriffen am 20.12.2023.

14 NT-proBNP: „N-terminal pro-B-type natriuretic peptide“ (Hormon, das als Herzinsuffizienzmarker dient).
